# Integrative taxonomy reveals a new species of *Callisto* (Lepidoptera, Gracillariidae) in the Alps

**DOI:** 10.3897/zookeys.473.8543

**Published:** 2015-01-20

**Authors:** Natalia Kirichenko, Peter Huemer, Helmut Deutsch, Paolo Triberti, Rodolphe Rougerie, Carlos Lopez-Vaamonde

**Affiliations:** 1 INRA, UR0633 Zoologie Forestière, F-45075 Orléans, France; 2 Sukachev Institute of Forest SB RAS, Akademgorodok 50/28, 660036, Krasnoyarsk, Russia; 3 Siberian Federal University, 79 Svobodny pr., 660041, Krasnoyarsk, Russia; 4 Naturwissenschaftliche Abteilung, Tiroler Landesmuseen Betriebsgesellschaft m.b.H., Feldstr. 11a, A-6020 Innsbruck, Austria; 5 Bannberg 22, 9911 Assling, East Tyrol, Austria; 6 Museo Civico di Storia Naturale, Lungadige Porta Vittoria 9, I37129, Verona, Italy; 7 Museum National d'Histoire Naturelle, UMR7205 ISYEB, F-75005 Paris, France

**Keywords:** COI, DNA barcoding, histone H3, mitochondrial-nuclear discordance, leaf-mining moths, contact zone, new species

## Abstract

Europe has one of the best-known Lepidopteran faunas in the world, yet many species are still being discovered, especially in groups of small moths. Here we describe a new gracillariid species from the south-eastern Alps, *Callisto basistrigella* Huemer, Deutsch & Triberti, **sp. n.** It shows differences from its sister species *Callisto coffeella* in morphology, the barcode region of the cytochrome *c* oxidase I gene and the nuclear gene histone H3. Both *Callisto basistrigella* and *Callisto coffeella* can co-occur in sympatry without evidence of admixture. Two *Callisto basistrigella* specimens show evidence of introgression. We highlight the importance of an integrative approach to delimit species, combining morphological and ecological data with mitochondrial and nuclear sequence data. Furthermore, in connection with this study, *Ornix blandella* Müller-Rutz, 1920, **syn. n.** is synonymized with *Callisto coffeella* (Zetterstedt, 1839).

## Introduction

Lepidoptera – butterflies and moths – are one of the most well-documented insect orders, but it is estimated that thousands of species, especially small-sized ones inhabiting the tropics, are still awaiting formal description. The integration of genetic data into taxonomic studies, especially with the advance of DNA barcoding campaigns (the construction of libraries of DNA barcodes for identification), has revealed many cases of cryptic or overlooked species in the tropics (Janzen et al. 2009, 2012), but also in some of the most studied regions such as Europe (Mutanen et al. 2012a, b, c, 2013).

Leaf-mining micro-moths in the family Gracillariidae are no exception. A study based on the analysis of DNA barcodes recently revealed a considerable number of undescribed species in the Neotropical region (Lees et al. 2013). In Europe, the systematics of this family is relatively well known, with 23 genera and 260 species recorded (De Prins and De Prins 2014) with new species still being discovered and described (Laštůvka and Laštůvka 2006, 2012; Triberti 2007; Laštůvka et al. 2013).

**Figures 1–8.**
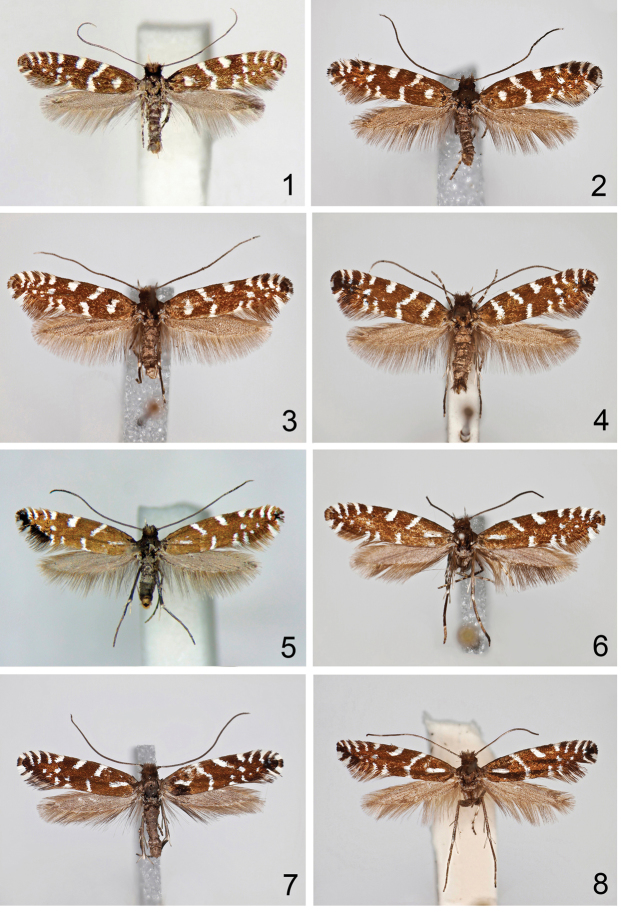
*Callisto* adults in dorsal view. **1** *Callisto coffeella*, male, Austria, Leitnertal, Oberer Stuckensee, 2150 m, 07.IX.2013, leg. Deutsch (PCHD) | voucher specimen № 3 | sample ID – NK318 | process ID CALCO003-14 **2** *Callisto coffeella*, male, Austria, Nordtirol, Bodenalpe, 2000 m, 9.–10.VII.1984, leg. Burmann (TLMF); **3** *Callisto coffeella*, male, Austria, Vorarlberg, Brandnertal, Böser Tritt, 1700-1800 m, 04.VII.1983, leg. Huemer (TLMF) **4** *Callisto coffeella*, female, Austria, Nordtirol, Obergurgl, 2000 m, e.l. M.III.1970, leg. Burmann (TLMF) **5** *Callisto basistrigella* sp. n., male, East Tyrol, Lienzer Dolomiten, Laserz, Dolomitenhütte, 1600 m, 12.VII.2013, leg. Deutsch (TLMF) | voucher specimen № 10 | sample ID – NK325 | process ID CALCO010-14 **6** *Callisto basistrigella* sp. n., male, Italy, Prov. Udine, Mte. Sernio, Forcella Nuviernulis, 1700 m, 16.VII.1988, leg. Huemer (TLMF) **7** *Callisto basistrigella* sp. n., male, Italy, Prov. Udine, Mt. Canin N, Rif. Gilberti, 1850–1950 m, 29.VII.2001, leg. Huemer (TLMF) **8** *Callisto basistrigella* sp. n., female, Italy, Prov. Udine, Montasio, 16.IX.1951, leg. Pinker (TLMF).

Here we focus on the gracillariid *Callisto coffeella* (Zetterstedt, 1839), an arctic-alpine species, which has been recorded from northern Europe, the Alps and a few other mountain areas of Europe. Its larvae initially mine leaves of several species of *Salix* and later feed in a folded leaf (Bengtsson and Johansson 2011). As all known *Callisto* species, *Callisto coffeella* adults have forewings with dark brown to blackish ground color with silvery white, oblique streaks (Figs 1–4). Due to these conspicuous wing markings they are relatively easy to identify. The alpha taxonomy of European *Callisto* has been established for a long time, with *Callisto insperatella* (Nickerl, 1864) being the most recently described species.

In a recent DNA barcoding study, Huemer (2011) found two genetic lineages within *Callisto coffeella*: one lineage formed by Austrian individuals from northern and central Alps, and a second one consisting of Italian specimens from the Southern Alps. Members of these two lineages differ on the basal silvery line of the forewings, which is transverse in south-eastern Alpine populations but vertical in all other examined populations (Fennoscandia, Northern and Central Alps). However, the author in contrast to other morphologically well-defined congeners found no differences in male and female genitalia. On the basis of phenotypical and genetic differences, it was suggested by P. Huemer that the south-eastern Alpine populations might represent a different subspecies.

Here we present new genetic, distribution and morphological data that support the hypothesis that individuals of *Callisto coffeella* from the south-eastern Alps represent a distinct lineage that we formally describe as a new species – *Callisto basistrigella* Huemer, Deutsch & Triberti, sp. n.

## Materials and methods

### Collections

Specimens examined in this study were obtained by rearing adults from leaf mines and by collecting adults flying by day around *Salix* bushes, mainly *Salix glabra* Scop., 1772 and *Salix waldsteiniana* Willd., 1806, but also a few *Salix appendiculata* Villars, 1789 and *Salix hastata* L., 1753. Some adults were collected at light trap or flew in the early morning hours. Data for all specimens studied morphologically and genetically can be found in the Suppl. material 1: Table S1.

### Morphology

We examined the morphology of 135 dried, pinned and mostly set specimens belonging to *Callisto coffeella* s.l., the majority originating from the Alps, and half a dozen from Scandinavia. Pinned specimens were photographed with an Olympus E 3 digital camera and an Olympus SZX 10 binocular microscope, and processed with Helicon Focus 4.3 software, resulting in multiple images. Images were later edited by using Adobe Photoshop Lightroom 2.3 software. Genitalia were photographed with an Olympus E1 digital camera through an Olympus BH2 microscope.

Genitalia dissections and slide mounts followed Robinson (1976). Morphometric analysis was carried out on genital preparations of 16 adult males (5 from the south-eastern alpine populations and 11 from Northern and Central alpine populations). Seven parameters were measured: phallus, valva, saccus, anellus and anellus process lengths, valva width and valva constriction.

All measurements were done on a Leica M 165C stereomicroscope by P. Triberti and expressed in mm. The dataset resulting from these measurements was analyzed using a multivariate approach – one-way ANOVA (Montgomery 2001), with species as a single categorical independent variable and the seven dependent measurement length variables mentioned above. Significance of each genital parameter was analyzed using a non-parametric Mann-Whitney test (MWT). Since our sampling size was rather small, particularly for southern populations, MWT was used because it does not require the normality of the data and allows tied values (Hollander and Wolfe 1999). With MWT, we tested the null hypothesis of no morphological differences. To avoid inter-correlations between dependent variables, we first estimated residual values of the correlated parameters using similar linear transformations (Draper and Smith 1998). We used this procedure for valva, saccus, and anellus process lengths, which were strongly correlated with phallus length. We used STATISTICA 8.0 (Stat Soft. Inc., USA) to conduct the analyses.

### DNA sequence analysis

DNA extracts were prepared from a single hind leg removed from each of 21 specimens of *Callisto coffeella* s.l. DNA extraction, PCR amplification and sequencing of the barcode region were carried out at the Canadian Centre for DNA Barcoding (CCDB, Biodiversity Institute of Ontario, University of Guelph) following standard protocols (De Waard et al. 2008). In addition, 14 samples were processed at INRA (Orléans, France). DNA was extracted using QIAGEN DNeasy Blood & Tissue Kit according to the manufacturer’s protocol. The COI barcoding fragment, 658 bp, was amplified via PCR using the primers LCO (5’ GGT CAA CAA ATC ATA AAG ATA TTG G 3’) and HCO (5’ TAA ACT TCA GGG TGA CCA AAA AAT CA 3’) and following standard conditions for the reaction (Folmer et al. 1994). PCR products were purified using the QIAGENAquick PCR purification kit and after used for the cycle sequencing reaction with Big Dye 3.1 (25 cycles of 35 min at 94 °C, 30 min at 46 °C and 1 min 30 sec at 72 °C).

Furthermore, 21 samples with DNA barcodes were also sequenced for the nuclear gene histone H3, a ~350 bp fragment, at INRA, Orléans. PCR for this gene was performed using primers Hex AF (5' -ATG GCT CGT ACC AAG CAG ACG GC -3') and Hex AR (5' -ATA TCC TTG GGC ATG ATG GTG AC-3') (Svenson and Whiting 2004) for 40 cycles (1 min at 94 °C, 1 min at 45 °C, 1 min at 65 °C and 10 min at 65 °C). Sequencing was carried out using a 3100 ABI genetic analyzer (Hitachi) with Big Dye 3.1 (25 cycles of 10 min at 96 °C, 5 min at 50 °C, 4 min at 60 °C). Both COI and histone H3 sequences were aligned using CodonCode Aligner 3.7.1. (CodonCode Corporation).

Sequence divergences were quantified using the Kimura 2-parameter model implemented within the analytical tools on BOLD (www.boldsystems.org) (Ratnasingham and Hebert 2007). A neighbor-joining (NJ) tree was constructed with MEGA 5.05 (Tamura et al. 2011). As a reference and to visually root the tree, we used one specimen of *Callisto insperatella* (Nickerl, 1864) (GRPAL094-10) for the COI tree and one specimen of *Parornix betulae* (Stainton, 1854) (GRACI621-10) for the histone H3 tree.

### Specimen and sequence information

Details on the collecting data for each specimen, as well as a photograph of vouchers, sequence records, trace files, and primer sequences used for PCR amplification, together with GenBank accession numbers are available through the following dataset (http://dx.doi.org/10.5883/DS-CALLISTO) in BOLD (www.boldsystems.org).

### Specimen depositories

LMK Landesmuseum Kärnten; Klagenfurt, Austria.

MCSN Museo Civico di Storia Naturale, Verona, Italy.

MCSNB Museo Civico di Scienze Naturali “E. Caffi”, Bergamo, Italy.

SMNK Staatliches Museum für Naturkunde, Karlsruhe, Germany.

TLMF Tiroler Landesmuseum Ferdinandeum, Innsbruck, Austria.

UO University of Oulu, Finland.

VND inatura Erlebnis Naturschau Dornbirn, Austria.

ZSM Zoologische Staatssammlung, Munich, Germany.

### Private collections

PCHD Helmut Deutsch, Bannberg, Assling, Tyrol, Austria.

PCJR Jurij Rekelj, Kranj, Slovenia.

PCJS Jürg Schmid, Illanz, Switzerland.

PCJW Josef Wimmer, Steyr, Austria.

PCJWdP Jurate and Willy De Prins, London, UK.

PCSG Stanislav Gomboc, Slovenia.

## Results

### Morphology

Morphological analysis of the 135 specimens confirms the differences observed in wing pattern in the south-eastern alpine population. Eighty-two of these individuals were diagnosed as *Callisto coffeella* and 53 as the new species *Callisto basistrigella*. In addition, we detected two moths which morphologically corresponded to *Callisto basistrigella* but with a COI barcode they fell into the cluster of *Callisto coffeella* (see below Molecular divergences).

#### Callisto coffeella

Taxon classificationAnimaliaLepidopteraGracillariidae

(Zetterstedt, 1839)

Oecophora coffeella Zetterstedt 1839: 1009.
Oecophora interruptella Zetterstedt 1839: 1009 [synonymised by Benander 1940: 61].
Ornix caelatella Zeller 1847: 585–586 [synonymised with *Oecophora interruptella* Zetterstedt, 1839 by Wocke (1862: 243)].
Ornix blandella Müller-Rutz 1920: 343. syn. n.
Annickia alpicola Gibeaux 1990: 23. [synonymised by Huemer 1990: 133].

##### Remarks.

*Oecophora coffeella* was described from an unspecified number of male specimens collected on the 14^th^ of July near Bjerkvik [according to original description ´Bjoerkvik” in Norwegian Lappland] (Zetterstedt 1839). *Oecophora interruptella* was described on the same page from a single male collected in 1836 in the Swedish province Dalarna, i.e. Dalecarlia by Boheman and from a female collected on 22^nd^ of July 1812 near Gibostad, i.e. Giebostad, Norway. The type material was examined and figured by Benander (1940) who synonymized both taxa.

*Annickia alpicola* was described from a single male specimen collected in the French Alps (Gibeaux 1990) and later synonymized with *Callisto coffeella* by Huemer (1990).

*Ornix caelatella* was described from a single male collected in Montenero (Tuscany, Italy) in May by Josef Mann (Zeller 1847), later this species was synonymized with *Ornix interruptella* (= *Callisto coffeella*) by Wocke (1862). The whereabouts of the holotype is unknown but the detailed original description and the Mediterranean locality disagree with both *Callisto coffeella* and *Callisto basistrigella*. However, a further specimen from Styria (Austria), later determined by Zeller (1850) as *caelatella* but defined as a particular form, may be conspecific with *Callisto coffeella*. We conclude that *Ornix caelatella* is a dubious taxon until the holotype will be rediscovered.

*Ornix blandella* was described by Müller-Rutz (1920) from a specimen bred by Paul Weber in Parpan (Switzerland) at 1500 m on *Salix* sp. Despite a focused search carried out by one of the authors (P. Triberti), the types were not found. However it was possible to study the original Müller-Rutz watercolours preserved in Naturhistorisches Museum Basel (Nr. 159 and 522) and they fully agree with typical *Callisto coffeella*. On the basis of what we conclude that *Ornix blandella* Müller-Rutz is a new synonym of *Callisto coffeella* Zetterstedt.

##### Description.

Adult (Figs 1–4). Head dark brown, with distinct dark brown tuft of raised scales on vertex, frons lighter, greyish brown, labial palp cream. Wingspan 10–12 mm; forewing dark brown with distinct whitish silvery markings: transverse oblique sub-basal line showing sexual dimorphism, well developed from costa to fold in female (Fig. 4), shorter in male (Figs 1–3) and not extending to costa, rarely reduced to a spot in fold; angulate fascia at one third frequently separated into costal and tornal line; costa furthermore with short median strigula and two pairs of distal strigulae; dorsum with two small distal spots; small discal spot, supplemented by up to 2-3 spots distally; particularly distomedial spots silvery rather than whitish silvery; fringes with distinct cilia line, basal half darker than distal half, termen with two whitish spots; hindwing grey-brown with same-colour fringes.

**Figures 9–12.**
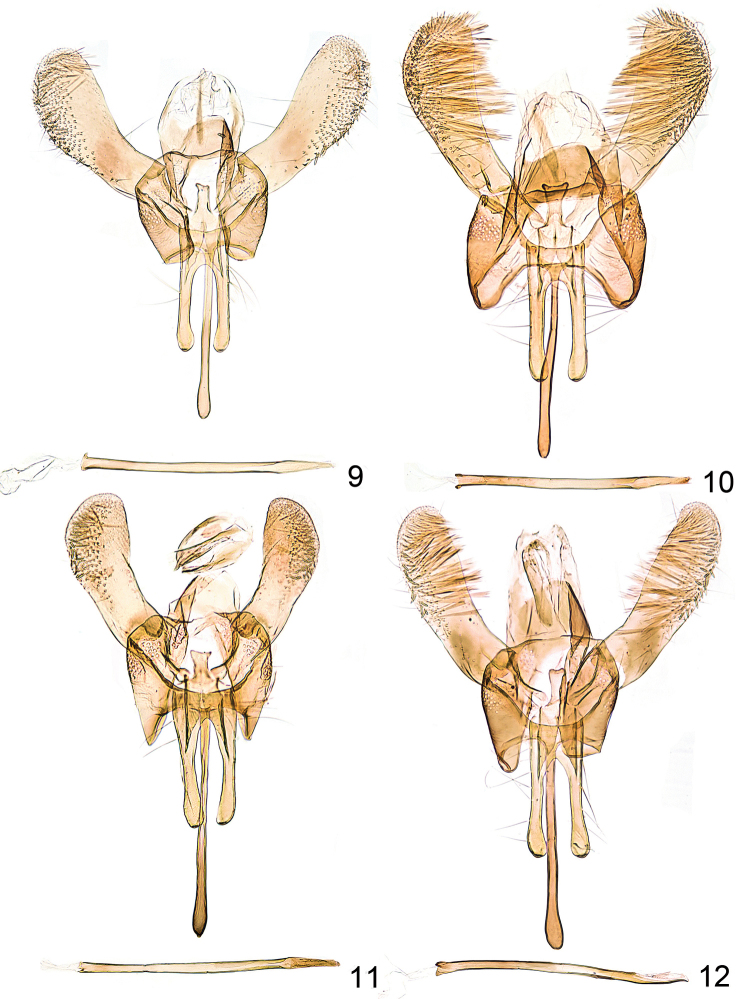
*Callisto*, male genitalia. **9** *Callisto coffeella*, Vorarlberg Zürs, 1800 m, 29.VI.1939, leg. Burmann, gen. slide TIN 1 (TLMF) **10** *Callisto coffeella* Teriol sept., Vent 2000 m, e.l. 01.III.1956, leg. Burmann, gen. slide TIN 4 (TLMF) **11** *Callisto basistrigella* sp. n., Italia sept. Prov. Udine, Mte. Sernio, Forcella Nuviernulis 1700 m, 16.VII.1988 leg. Huemer gen. slide TIN 2 (TLMF) **12** *Callisto basistrigella* sp. n. Italia sept. Prov. Udine, Mte. Sernio, Forcella Nuviernulis 1700 m, 16.VII.1988 leg. Huemer gen. slide TIN 3(TLMF).

**Figures 13–16.**
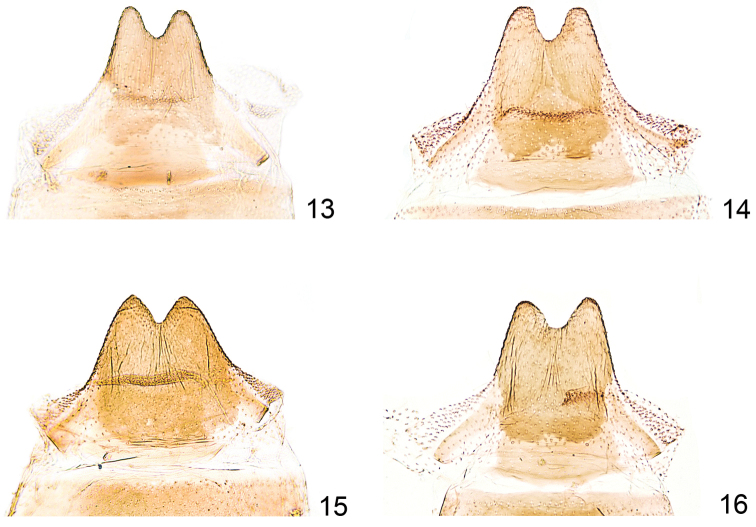
*Callisto*, male, segment 8. **13** *Callisto coffeella*, Vorarlberg Zürs, 1800 m, 29.VI.1939, leg. Burmann, gen. slide TIN 1 (TLMF) **14** *Callisto coffeella*, Teriol sept., Vent 2000 m, e.l. 01.III.1956, leg. Burmann, gen. slide TIN 4 (TLMF) **15** *Callisto basistrigella* sp. n., Italia sept. Prov. Udine, Mte. Sernio, Forcella Nuviernulis 1700 m, 16.VII.1988, leg. Huemer, gen. slide TIN 2 (TLMF) **16** *Callisto basistrigella* sp. n., Italia sept. Prov. Udine, Mte. Sernio, Forcella Nuviernulis 1700 m, 16.VII.1988 leg. Huemer gen. slide TIN 3 (TLMF).

Genitalia and eighth segment male (Figs 9–10, 13–14). Sternite 8 projected, bilobed. Tuba analis with long and thin subscaphium; valva slender, distally widened, with evenly rounded apex; vinculum laterally projected; saccus long and slender, rod-like, about as long as valva; anellus with pair of long and projecting processes; phallus slender, straight, about twice as long as valva, without distinct modifications, apically pointed.

**Figures 17–18.**
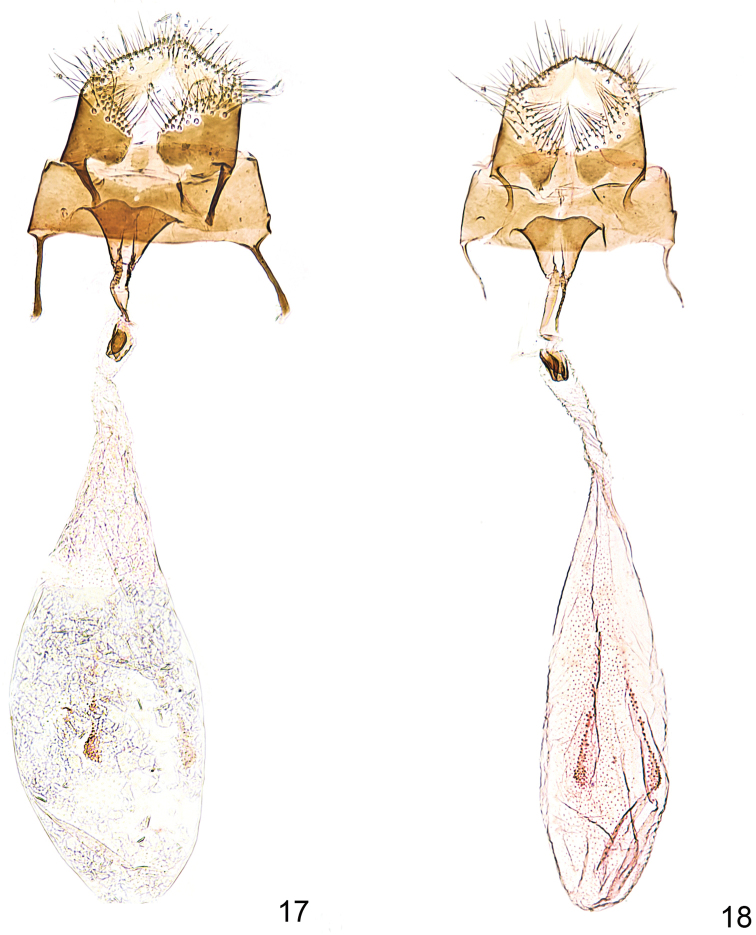
*Callisto*, female genitalia. **17** *Callisto coffeella*, Austria, Vorarlberg, Brandnertal, Böser Tritt, 1700–1800 m, 02.VII.1983, leg. Huemer gen. slide TIN 9 (TLMF) **18** *Callisto basistrigella* sp. n., Prov. Udine, Montasio, 16.IX.1951, leg. Pinker gen. slide TIN 8 (TLMF).

Genitalia female (Fig. 17). Apophyses posteriors shorter than anteriores; segment 8 short, bare, intersegmental membrane to papillae anales very reduced; sterigma simple with ostium bursae wide, ventral margin medially more or less indented; antrum cup-shaped; ductus bursae moderately long and smooth, short sclerite just before antrum; corpus bursae, oval, longer than ductus bursae, signa formed by scobinations arranged in two longitudinal bands.

##### Distribution.

The species is restricted to higher mountain areas and shows an arctic-alpine distribution pattern. According to various publications (i.e. Bengtsson and Johansson 2011, Heath and Emmet 1985, Huemer and Tarmann 1993, SwissLepTeam 2010) the species is locally distributed in the central and northern parts of Scandinavia, northern Scotland, and in the eastern, northern and central Alps. Most of these regions were included in our study, particularly alpine regions of Italy, Austria, Switzerland and Slovenia; sampling was also done in southeast of Germany and in Scandinavia (Norway, Sweden, Finland). In the Southern Alps it is known from a single record in France and from Aosta Valley to Carnic Alps in Italy. *Callisto coffeella* is also reported from Western Russia (Sinev 2008), Ukraine, Poland, Slovakia, and United Kingdom (De Prins and De Prins 2014) but we have been unable to check material from these countries.

##### Bionomics.

The larval stage feeds on various species of mountainous *Salix* such as *Salix arbuscula* L., 1753 (which may refer to *Salix arbuscula* in northern Europe or *Salix waldsteiniana* in Central Europe), *Salix phylicifolia* L., 1753 (Heath and Emmet 1985), *Salix repens* L., 1753 (syn: *Salix fusca*), *Salix myrsinifolia* Salisb., 1796, *Salix silesiaca* Willd., (1806) [basionym] (De Prins and De Prins 2014). In our study, *Callisto coffeella* was also reared from *Salix glabra*. Initially the larva produces a short epidermal gallery which suddenly widens to a blotch tentiform mine on the lower surface of a leaf, similar in appearance to mines of the genus *Phyllonorycter*. Later the mine is vacated and the larva forms a shelter along a leaf margin, folding an edge downwards as in many *Parornix*. Pupation takes place in a cocoon on the branch of the host-plant or in the laboratory between leaf litter and tissue. Hibernation occurs in the pupal stage. The adult is on the wing in June and July. It can be found during the day, most frequently in the morning and early evening flying around the hostplant. The species lives in montane and subalpine habitats of the dwarf-shrub zone both on calcareous and siliceous soil.

#### Callisto basistrigella

Taxon classificationAnimaliaLepidopteraGracillariidae

Huemer, Deutsch & Triberti sp. n.

http://zoobank.org/95B2011C-A39A-436E-8FF4-35ABEE5827E1

##### Type material.

Holotype (Fig. 5): 1 male, East Tyrol, Lienzer Dolomiten, Laserz, Dolomitenhütte, 1600 m, 12.VII.2013, leg. Deutsch (TLMF) | voucher specimen № 10 | sample ID – NK325 | process ID CALCO010-14.

##### Paratypes.

33 males and 11 females.

Austria: 3 males, East Tyrol, Lienzer Dolomiten, Lavanter Almtal, 1200-1400 m, 07.VI.1998, leg. Deutsch (TLMF); 1 male, East Tyrol, Lienzer Dolomiten, Laserzgebiet, 1800-2000 m, 21.VI.1999, leg. Deutsch (TLMF); 1 male, East Tyrol, Carnic Alps, Leitnertal, Oberer Stuckensee, 2150 m, 14.VII.2013, leg. Deutsch (PCHD) | voucher specimen № 8 | sample ID – NK323 | process ID CALCO008-14; 2 males, East Tyrol, Carnic Alps, Leitnertal, Oberer Stuckensee, 2150 m, 07.IX.2013, leg. Deutsch (PCHD) | voucher specimens № 1 and № 2 | sample IDs – NK316 and NK317 | process Ids CALCO001-14 and CALCO002-14; 1 female, East Tyrol, Lienzer Dolomiten, Hochstadel, 2000 m, VII.1952, leg. Pinker (TLMF); 2 females, East Tyrol, Carnic Alps, Leitnertal, Oberer Stuckensee, 2150 m, 07.IX.2013, leg. Deutsch (PCHD) | voucher specimens № 4 and № 6 sample | sample IDs – NK319 and NK321 | process Ids CALCO004-14 and CALCO006-14.

Italy: 4 males, Prov. Belluno, Passo di Valparola E, 2200-2300 m, 20.VII.2009, leg. Huemer (TLMF); 1 female, same data but gen. slide TRB3893 and BC TLMF Lep 01801 (TLMF); 1 male, A. Carniche, Sappada, Casera Sesis, 1800 m, 12.VI. unknown year, leg. Rocca, gen. slide TRB 1778 (MCSN); 1 male, A. Carniche, Sappada, Passo Siera, 1600 m, 04.VII.1933, leg. Rocca, gen. slide TRB 1785 (MCSN); 1 male, A. Carniche, Sappada, Hosthaus, 1800 m, 14.VII.1936, leg. Rocca (MCSN); 2 males, 2 females, A. Carniche, Sappada, L. d’Olbe, 2000 m, 02.VII.1933, leg. Rocca, gen. slide TRB284 male, TRB3894 male (MCSN); 1 male, Prov. Udine, Mte. Sernio-Massiv Forcella Nuviernulis 1700 m, 16.VII.1988, leg. Huemer, GU TIN2 male P. Huemer ’Callisto coffeella Zett. det. Triberti’ (TLMF); 1 male, Prov. Udine, Mte. Sernio-Massiv Forcella Nuviernulis 1700 m, 16.VII.1988, leg. Huemer, GU TIN3 male (TLMF); 1 male, 1 female, Prov. Udine, Montasio, 16.IX.1951, leg. Pinker, gen. slide TIN8 female (TLMF); 11 males, 1 female, Prov. Udine, Monte Canin N, Rif. Gilberti Umg., 1850-1950 m, 29.VII.2001, leg. Huemer (TLMF); 1 male, 1 female, Prov. Udine, Monte Canin, Biv. Marussich, 2040 m, 06.VII.2002, leg. Wieser (LMK); 3 males, Prov. Udine, Monte Canin, Sella di Grubia, 1700 m, 20.VI.2003, leg. Wieser (LMK).

Slovenia: 1 female, Crna Prst, 1400 m, 18.VII.1899, leg. Penther (TLMF).

##### Diagnosis.

In external appearance *Callisto basistrigella* is distinguishable from *Callisto coffeella* by its forewing pattern. In *Callisto basistrigella*, the sub-basal whitish silvery line of the forewing is almost parallel and lies in the fold, whereas in *Callisto coffeella* this line is transverse to the wing axis or reduced to a spot. On average, the forewings are slightly narrower than in *Callisto coffeella* (visible in series). Sexual dimorphism, as observed in *Callisto coffeella*, is absent in *Callisto basistrigella*. Genitalia do not provide obvious diagnostic differences but the length of the phallus is significantly longer in *Callisto basistrigella* than in *Callisto coffeella* although more specimens would be needed to confirm this difference (see Genital morphometrics).

##### Description.

Adult (Figs 5–8). Wingspan 10.5–13.0 mm; forewing in sub-basal area with longitudinal, slightly oblique, whitish silvery line in fold. Other characters as described above for *Callisto coffeella*. The angulate fascia at one third of forewing frequently separated into costal and tornal line.

Genitalia and subgenital segments male (Figs 11–12, 15–16). As described above for *Callisto coffeella*.

Genitalia female (Fig. 18). As described above for *Callisto coffeella*.

##### Distribution.

**Figure 19.**
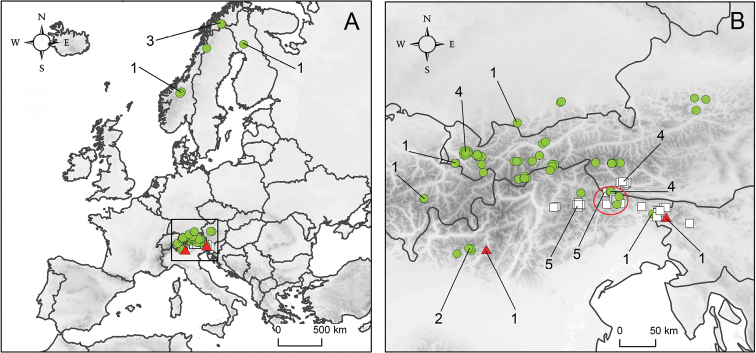
**A** sampling area of *Callisto coffeella* and *Callisto basistrigella* in Europe. **B** close up of the distribution of *Callisto coffeella* (green circles) and *Callisto basistrigella* (white squares) in the Alps; two *Callisto basistrigella* specimens (red triangles) show evidence of introgression. On Figs 19A, 19B, the 35 barcoded specimens are shown with numbers (1-5). The red circle on Fig. 19B shows the contact zone where both species occur together (Leitnertal, Eastern Tyrol, Austria and Sappada, Italy). When several samples were investigated per locality, the samples with the same coordinates have been slightly shifted in order to visualize overlapping data points on Fig. 19B.

Only known from a small area in the south-eastern Alps, ranging from the Dolomites (Italy) in the west to the Julian Alps (Slovenia) in the east and the Carnic Alps and Lienzer Dolomiten (Austria) in the north (Fig. 19A, B).

##### Etymology.

The name refers to the characteristic wing markings.

##### Bionomics.

Early stages are undescribed. Both *Callisto basistrigella* and *Callisto coffeella* adults have been collected during the day, flying around low bushes of alpine *Salix glabra* and *Salix waldsteiniana*. The flight period is largely dependent on exposure and snow coverage and usually extends between early June and late July. Under extreme conditions such as harsh winters adults have been collected as late as mid-September. The habitats are related to the dwarf-shrub zone and include subalpine meadows, rock formations and scree with *Salix*-bushes and shrubs. *Callisto basistrigella* is restricted to limestone with an altitudinal range from about 1200 to 2300 m.

##### Genital morphometrics.

**Figure 20.**
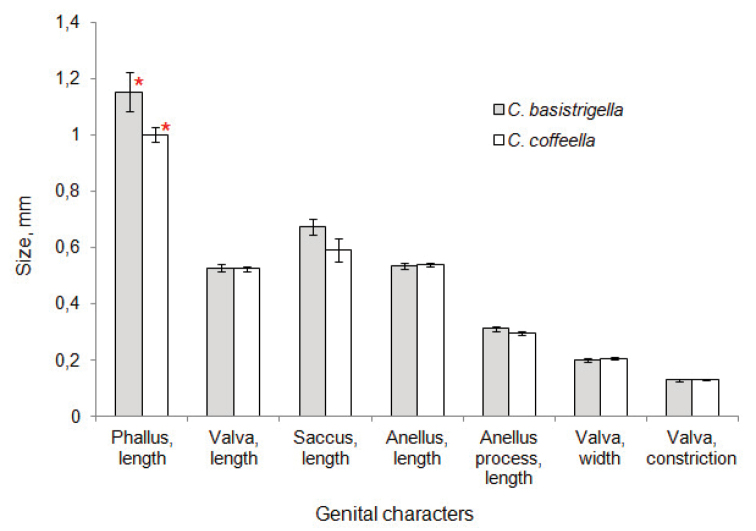
Genitalia measurements (mean values ± standard error) for the two *Callisto* species studied. The bars marked by an asterisk are significantly different from each other (MWT: *Z* = 2.36, *N* = 16, *p* = 0.02); in others cases, there is no difference between the species.

Multivariate ANOVA analysis based on morphometric of seven genital characteristics of the male moths failed to find any significant difference between *Callisto coffeella* and *Callisto basistrigella* (*Wilks’ λ* = 0.36, *F* = 2.07, *p* = 0.16). Six out of seven parameters, i.e valva, saccus, anellus and anellus process lengths, valva width and valva constriction were not found to differ in the two species. Non-parametric Mann-Whitney test however indicated that the phallus is significantly longer in *Callisto basistrigella* than in *Callisto coffeella* (MWT: *Z* = 2.36, *N* = 16, *p* = 0.02), although sample sizes remain relatively small (*Callisto basistrigella* N = 5, *Callisto coffeella* N = 11) (Fig. 20). Two specimens of *Callisto basistrigella* from Sappada (Italy) made significant contributions to phallus length value of the species, exceeding the averaged length of *Callisto coffeella* phallus by 27%.

### Molecular divergences

**Figure 21.**
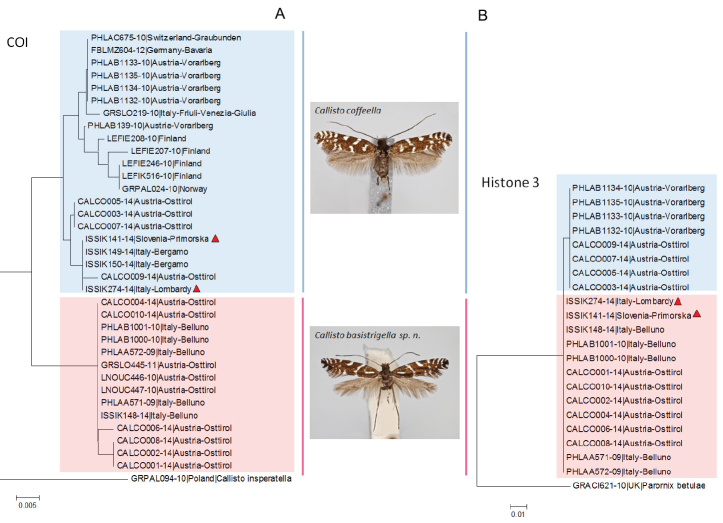
**A** neighbor joining tree based on the COI barcode fragment and **B** based on the histone H3 gene. The two specimens (ISSIK141-14, ISSIK274-14) with the *Callisto basistrigella* phenotype, but branching within the *Callisto coffeella* DNA barcode and within the *Callisto basistrigella* histone H3 cluster are marked with red triangles (as in Fig. 19) in both trees.

**DNA barcodes.** We obtained DNA barcodes for 21 specimens of *Callisto coffeella* and 14 specimens of *Callisto basistrigella*. Their analysis revealed that the samples of these species form two distinct clusters in the NJ tree (Fig. 21A), with two exceptions: one Slovenian (ISSIK141-14) and one Italian (ISSIK274-14) identified morphologically (and also by nuclear data, see below) as *Callisto basistrigella* grouped with *Callisto coffeella* (Fig. 21A), suggesting introgression or incomplete lineage sorting.

Excluding these two records, pairwise interspecific distances range between 1.39% and 2.37%, with a mean value of 1.75% (*sd* = 0.2). Within *Callisto basistrigella* and *Callisto coffeella*, respectively, genetic distances range from 0 to 0.31% (mean-value 0.17%, *sd* = 0.11) and from 0 to 1.23% (mean-value 0,56%, *sd* = 0.31). Sequence comparison revealed eight diagnostic substitutions (Table 1).

**Table 1.** Diagnostic substitutions in COI-DNA barcode sequences of *Callisto coffeella* and *Callisto basistrigella*.

Position	70	88	145	206	271	295	547	631
*Callisto basistrigella*	T	T	T	C	T	A	A	A
*Callisto coffeella*	A	A	C	T	C	C	C	G

**Histone H3.** We obtained sequences of the nuclear gene histone H3 (328 bp) for the same 21 moths that were barcoded. H3 showed a high conservatism, with a single diagnostic nucleotide substitution at position 151, dividing the studied specimens into two clusters matching exactly the morphology-based separation of *Callisto coffeella* and *Callisto basistrigella* (Fig. 21B).

The Slovenian (ISSIK141-14) and Italian (ISSIK274-14) specimens, morphologically assigned to *Callisto basistrigella* and whose DNA barcodes clustered within *Callisto coffeella* (Fig. 21A), have histone H3 sequences identical to other *Callisto basistrigella* specimens (Fig. 21B).

**Contact zone.** Both *Callisto basistrigella* and *Callisto coffeella* were found to occur in the same localities in the Carnic Alps (Leitnertal, Eastern Tyrol, Austria) at the altitude up to 2150 m (Fig. 19). Out of 9 specimens collected in Leitnertal (1 *Callisto coffeella*, 1 *Callisto basistrigella* collected on 14.VII.2013, 2 *Callisto basistrigella* on 27.VII.2013, about 30 leaf mines on *Salix glabra* on 07.IX.2013), 4 specimens were identified based on both morphology and genetic data as *Callisto coffeella* and 5 specimens were identified as *Callisto basistrigella*. In addition, 7 of 9 samples (i.e. 3 specimens of *Callisto coffeella* and 4 of *Callisto basistrigella*) were reared from the same host plant – *Salix glabra*. Furthermore old records confirm this sympatry in the nearby Italian Carnic Alps, in the surroundings of Sappada (1 *Callisto coffeella* and 1 *Callisto basistrigella* were collected in Passo Siera, 1600 m, 04.VII.1933; 1 *Callisto coffeella* and 4 *Callisto basistrigella* – in L. d’Olbe, 2000 m, 02.VII.1933) (Fig. 19B). No evidence of genetic admixture was detected in the contact zone.

## Discussion

Our study used newly generated mitochondrial and nuclear data in combination with morphological and morphometric data to characterize the variability of *Callisto coffeella* across its range. We confirmed the existence of two distinct lineages, one of which is described here as *Callisto basistrigella*. Its status as a distinct species is supported by morphology, nuclear DNA (histone H3 gene) and by mtDNA (COI-DNA barcodes), although shared haplotypes of the latter suggest introgression or incomplete lineage sorting.

**Species delineation with DNA barcodes.** In Lepidoptera, although authors generally reject the use of a threshold to delineate species, an empirical 2% (K2P) intraspecific distance value has often been proposed, pragmatically, as indicating “deep divergence” suggestive of potential overlooked or cryptic diversity (Hebert et al. 2010; Hausmann et al. 2011; Huemer et al. 2014; Rougerie et al. 2014). In the present study, we brought to the fore a case of overlooked species in which the DNA barcode divergence between the newly recognized pair of species can be as low as 1.39%; this case would then have been missed if the screening of our results had been based on the strict application of a 2% threshold before triggering further investigation. Furthermore, we reported two cases of nuclear/mitochondrial discordance in samples ISSIK141-14 and ISSIK274-14 (see Fig. 21) where histone H3 sequences and morphology conflict with the assignment based on DNA barcodes. This may have been caused by genetic introgression or incomplete lineage sorting. This finding is important as it highlights the necessary caution when using DNA barcodes for the identification of this and other pairs of closely related species. Whereas most specimens are likely to be correctly identified on the basis of this genetic marker (discordance was detected in two (5.7%) out of 35 specimens only), one should use characters of the wing pattern (or additional genetic data) to confirm identities where certain identification is needed.

**Contact zone.** We found that *Callisto basistrigella* occurs in sympatry with *Callisto coffeella* in the Carnic Alps, Leitnertal, 2150 m (East Tyrol, Austria) and Sappada 1600-1800 m (Italy), without evidence of admixture in this area. The two cases of nuclear/mitochondrial discordance revealed suggests possible genetic introgression between the two species. Further sampling and the use of fast evolving markers will be needed to investigate the course of a putative contact zone as well as the extent of gene flow between the two species.

**Biogeography and speciation.** The distribution of *Callisto basistrigella* as currently known is shared by several other endemic Lepidoptera. The south-eastern Alps is considered as one of the major areas of endemism in the region (Huemer 1998). However, most of the taxa restricted to this area have been defined only by morphological characters so far and their taxonomy has to be re-assessed using molecular data. The specific distinctness of *Udea murinalis* (Fischer von Röslerstamm, 1842) and the allopatric south-eastern alpine *Udea carniolica* Huemer & Tarmann, 1989 (Lepidoptera, Crambidae) both separated by moderate morphological differences, was recently well supported by molecular datasets (Mally and Nuss 2011). Another alleged set of sister taxa include *Dichrorampha bugnionana bugnionana* (Duponchel, 1843) and the south-eastern alpine subspecies *Dichrorampha bugnionana dolomitana* Huemer, 1993 with a significant barcode divergence (Huemer unpublished data).

Allopatric isolation during the last glacial period is probably the main process by which *Callisto basistrigella* and *Callisto coffeella* diverged. Indeed, as many other cold-adapted Lepidoptera *Callisto coffeella* populations may have had a wide distribution in the periglacial tundra belts during the last glacial period. With increasing temperatures during the last interglacial period, *Callisto coffeella* may have moved northwards while southern populations moved up in altitude in the Alps (Mutanen et al. 2012c). On the other hand, *Callisto basistrigella* is restricted to the south-eastern Alps and may have derived from populations having occupied distinct refugia during the last glacial period.

Our results highlight the need to carry out additional intraspecific studies looking at patterns of both morphological and genetic variability within species across their ranges, which can reveal overlooked diversity and new species (Huemer 2011, Huemer and Mutanen 2012, Mutanen et al. 2012a, b, c), in regions that are thought to be well studied.

## Supplementary Material

XML Treatment for Callisto coffeella

XML Treatment for Callisto basistrigella

## Supplementary materials

Supplementary material 1 List of studied specimens of *Callisto coffeella* and *Callisto basistrigella* and collection data. Data type: collection data / voucher depository / genetic code. Explanation note: The list of 135 examined specimens of *Callisto coffeella* and *Callisto basistrigella* sp. n., their collection data (country, locality, GPS coordinates, collection date and collector name) and depository data (museum or private collection) are provided in the supplementary table S1. All specimens have been studied morphologically; the barcoded samples are supplied with sample ID, process ID, GenBank COI and GenBank H3 (if nuclear gene histone H3 was analyzed). This dataset is made available under the Open Database License (http://opendatacommons.org/licenses/odbl/1.0/). The Open Database License (ODbL) is a license agreement intended to allow users to freely share, modify, and use this Dataset while maintaining this same freedom for others, provided that the original source and author(s) are credited.Natalia Kirichenko, Peter Huemer, Helmut Deutsch, Paolo Triberti, Rodolphe Rougerie, Carlos Lopez-Vaamonde
