# Supplementary material for: Integrative taxonomy reveals a new species of Callisto (Lepidoptera, Gracillariidae) in the Alps
Source: Zookeys. 2015 Jan 20;(473):157–76. doi: 10.3897/zookeys.473.8543 (PMC4304045; doi:10.3897/zookeys.473.8543)
Supplement: Supplementary material 1 — List of studied specimens of Callisto coffeella and Callisto basistrigella and collection data. [file zookeys-473-157-s001.pdf]

Supplementary material

Table S1. List of studied specimens of *Callisto coffeella* and *C. basistrigella* and collection data*

№	Species name[1]	Sample ID	Process ID	Genbank COI	Genbank H3	Collector	Collection[2]	Collection date	Country	Locality	Latitude	Longitude	Elevation (m)
1.	*C.b.*	CLV151010	LNOUC446-10	JF855560	-	Deutsch H.	PCHD	17.VI.2005	Austria	Lavanter Almtal	46.7747	12.8539	1500
2.	*C.b.*	CLV1511	LNOUC447-10	JF855561	-	Deutsch H.	PCHD	17.VI.2005	Austria	Lavanter Almtal	46.7747	12.8539	1500
3.	*C.b.*	CLV1965	GRSLO445-11	JF848501	-	Gomboc S.	PCSG	17.VI.2005	Austria	Lavanter Almtal	46.7920	12.8060	1400
4.	*C.b.*	CLV5633	ISSIK148-14	KM253785	KM253804	Huemer P.	TLMF	20.VII.2009	Italy	Passo di Valparola E	46.5255	11.9998	2200
5.	*C.b.*	NK316	CALCO001-14	KM253786	KM253805	Deutsch H.	PCHD	07.IX.2013	Austria	Leitnertal, Oberer Stuckensee	46.6648	12.5444	2150
6.	*C.b.*	NK317	CALCO002-14	KM253783	KM253801	Deutsch H.	PCHD	07.IX.2013	Austria	Leitnertal, Oberer Stuckensee	46.6648	12.5444	2150
7.	*C.b.*	NK319	CALCO004-14	KM253787	KM253808	Deutsch H.	PCHD	07.IX.2013	Austria	Leitnertal, Oberer Stuckensee	46.6648	12.5444	2150
8.	*C.b.*	NK321	CALCO006-14	KM253781	KM253799	Deutsch H.	PCHD	07.IX.2013	Austria	Leitnertal, Oberer Stuckensee	46.6648	12.5444	2150
9.	*C.b.*	NK323	CALCO008-14	KM253775	KM253795	Deutsch H.	PCHD	14.VII.2013	Austria	Leitnertal, Oberer Stuckensee	46.6648	12.5444	2150
10.	*C.b.*	NK325	CALCO010-14	KM253776	KM253796	Deutsch H.	TLMF	12.VII.2013	Austria	Dolomitenhuette	46.7910	12.7845	1600
11.	*C.b.*	TLMF Lep 00611	PHLAA571-09	HM425988	KM253790	Huemer P.	TLMF	20.VII.2009	Italy	Passo di Valparola E	46.5255	11.9998	2200
12.	*C.b.*	TLMF Lep 00612	PHLAA572-09	HM381450	KM253791	Huemer P.	TLMF	20.VII.2009	Italy	Passo di Valparola E	46.5255	11.9998	2200

№	Species name[1]	Sample ID	Process ID	Genbank COI	Genbank H3	Collector	Collection[2]	Collection date	Country	Locality	Latitude	Longitude	Elevation (m)
13.	*C.b.*	TLMF Lep 01800	PHLAB1000-10	HM381450	KM253791	Huemer P.	TLMF	20.VII.2009	Italy	Passo di Valparola E	46.5255	11.9998	2200
14.	*C.b.*	TLMF Lep 01801	PHLAB1001-10	HQ968272	KM253806	Huemer P.	TLMF	20.VII.2009	Italy	Passo di Valparola E	46.5255	11.9998	2200
15.	*C.b.*	-	-	-	-	Germanytsch H.	TLMF	21.VII.1999	Austria	Lz.Dol., Laserzgebiet	46.7699	12.7935	1800
16.	*C.b.*	-	-	-	-	Pinker	TLMF	01.VII.1952	Austria	Hochstadel	46.7600	12.8500	2000
17.	*C.b.*	-	-	-	-	Deutsch	TLMF	07.VI.1998	Austria	Lz.Dol., Lavanter Almtal	46.7747	12.8539	1200
18.	*C.b.*	-	-	-	-	Deutsch	TLMF	07.VI.1998	Austria	Lz.Dol., Lavanter Almtal	46.7747	12.8539	1200
19.	*C.b.*	-	-	-	-	Deutsch	TLMF	07.VI.1998	Austria	Lz.Dol., Lavanter Almtal	46.7747	12.8539	1200
20.	*C.b.*	-	-	-	-	Huemer P.	TLMF	16.VII.1988	Italy	Forcella Nuviernulis	46.4750	13.1473	1732
21.	*C.b.*	-	-	-	-	Pinker	TLMF	16.IX.1951	Italy	Montasch/ Raibl S	46.4500	13.5833	1600
22.	*C.b.*	-	-	-	-	Pinker	TLMF	16.IX.1951	Italy	Montasch/ Raibl S	46.4500	13.5833	1600
23.	*C.b.*	-	-	-	-	Huemer P.	TLMF	20.VII.2009	Italy	Passo di Valparola E	46.5255	11.9998	2200
24.	*C.b.*	-	-	-	-	Huemer P.	TLMF	20.VII.2009	Italy	Passo di Valparola E	46.5255	11.9998	2200
25.	*C.b.*	-	-	-	-	Huemer P.	TLMF	20.VII.2009	Italy	Passo di Valparola E	46.5255	11.9998	2200
26.	*C.b.*	-	-	-	-	Huemer P.	TLMF	20.VII.2009	Italy	Passo di Valparola E	46.5255	11.9998	2200
27.	*C.b.*	-	-	-	-	Huemer P.	TLMF	29.VII.2001	Italy	Rifugio Gilberti	46.3728	13.4628	1850
28.	*C.b.*	-	-	-	-	Huemer P.	TLMF	29.VII.2001	Italy	Rifugio Gilberti	46.3728	13.4628	1850

№	Species name[1]	Sample ID	Process ID	Genbank COI	Genbank H3	Collector	Collec-tion[2]	Collection date	Country	Locality	Lati-tude	Longi-tude	Eleva-tion (m)
29.	*C.b.*	-	-	-	-	Huemer P.	TLMF	29.VII.2001	Italy	Rifugio Gilberti	46.3728	13.4628	1850
30.	*C.b.*	-	-	-	-	Huemer P.	TLMF	29.VII.2001	Italy	Rifugio Gilberti	46.3728	13.4628	1850
31.	*C.b.*	-	-	-	-	Huemer P.	TLMF	29.VII.2001	Italy	Rifugio Gilberti	46.3728	13.4628	1850
32.	*C.b.*	-	-	-	-	Huemer P.	TLMF	29.VII.2001	Italy	Rifugio Gilberti	46.3728	13.4628	1850
33.	*C.b.*	-	-	-	-	Huemer P.	TLMF	29.VII.2001	Italy	Rifugio Gilberti	46.3728	13.4628	1850
34.	*C.b.*	-	-	-	-	Huemer P.	TLMF	29.VII.2001	Italy	Rifugio Gilberti	46.3728	13.4628	1850
35.	*C.b.*	-	-	-	-	Huemer P.	TLMF	29.VII.2001	Italy	Rifugio Gilberti	46.3728	13.4628	1850
36.	*C.b.*	-	-	-	-	Huemer P.	TLMF	29.VII.2001	Italy	Rifugio Gilberti	46.3728	13.4628	1850
37.	*C.b.*	-	-	-	-	Huemer P.	TLMF	29.VII.2001	Italy	Rifugio Gilberti	46.3728	13.4628	1850
38.	*C.b.*	-	-	-	-	Huemer P.	TLMF	29.VII.2001	Italy	Rifugio Gilberti	46.3728	13.4628	1850
39.	*C.b.*	-	-	-	-	Penther	TLMF	18.VII.1899	Slovenia	Crna prst	46.2378	13.9981	1400
40.	*C.b.*	-	-	-	-	Huemer P.	TLMF	6.VII.2008	Italy	Schlern, Touristensteig Dolomitfelswände schattig	46.5108	11.5897	2200
41.	*C.b.*	-	-	-	-	Huemer P.	TLMF	15.VIII.2006	Italy	SE Weisslahn, E Ums/ Völs am Schlern	46.5006	11.5508	1580
42.	*C.b.*	-	-	-	-	Rocca L.	MCSN	12.VI. unknown year	Italy	A. Carniche, Sappada, Casera Sesis	46.5901	12.7406	1800
43.	*C.b.*	-	-	-	-	Rocca L.	MCSN	04.VII.1933	Italy	A. Carniche, Sappada, Passo Siera	46.5433	12.6772	1600
44.	*C.b.*	-	-	-	-	Rocca L.	MCSN	14.VII.1936	Italy	A. Carniche, Sappada, Hosthaus	46.5228	12.5150	1800
45.	*C.b.*	-	-	-	-	Rocca L.	MCSN	02.VII.1933	Italy	A. Carniche, Sappada, L.	46.5929	12.6916	2000

№	Species name[1]	Sample ID	Process ID	Genbank COI	Genbank H3	Collector	Collec-tion[2]	Collection date	Country	Locality	Lati-tude	Longi-tude	Eleva-tion (m)
										d'Olbe			
46.	*C.b.*	-	-	-	-	Rocca L.	MCSN	02.VII.1933	Italy	A. Carniche, Sappada, L. d'Olbe	46.5929	12.6916	2000
47.	*C.b.*	-	-	-	-	Rocca L.	MCSN	02.VII.1933	Italy	A. Carniche, Sappada, L. d'Olbe	46.5929	12.6916	2000
48.	*C.b.*	-	-	-	-	Rocca L.	MCSN	02.VII.1933	Italy	A. Carniche, Sappada, L. d'Olbe	46.5929	12.6916	2000
49.	*C.b.*	-	-	-	-	Huemer P.	TLMF	16.VII.1988	Italy	Forcella Nuviernulis	46.4750	13.1473	1732
50.	*C.b.*	-	-	-	-	Wieser C.	LMK	VI.VII.2002	Italy	Prov. Udine, Monte Canin, Biv. Marussich	46.3672	13.4253	2040
51.	*C.b.*	-	-	-	-	Wieser C.	LMK	VI.VII.2002	Italy	Prov. Udine, Monte Canin, Biv. Marussich	46.3672	13.4253	2040
52.	*C.b.*	-	-	-	-	Wieser C.	LMK	20.VI.2003	Italy	Sella di Grubia	46.3656	13.4164	1700
53.	*C.b.*	-	-	-	-	Wieser C.	LMK	20.VI.2003	Italy	Sella di Grubia	46.3656	13.4164	1700
54.	*C.c.*	NK318	CALCO003-14	KM253770	KM253788	Deutsch H.	PCHD	07.IX.2013	Austria	Leitnertal, Oberer Stuckensee	46.6648	12.5444	2150
55.	*C.c.*	NK320	CALCO005-14	KM253777	KM253798	Deutsch H.	PCHD	07.IX.2013	Austria	Leitnertal, Oberer Stuckensee	46.6648	12.5444	2150
56.	*C.c.*	NK322	CALCO007-14	KM253784	KM253803	Deutsch H.	PCHD	07.IX.2013	Austria	Leitnertal, Oberer Stuckensee	46.6648	12.5444	2150
57.	*C.c.*	NK324	CALCO009-14	KM253774	KM253794	Deutsch H.	PCHD	14.VII.2013	Austria	Leitnertal, Oberer Stuckensee	46.6648	12.5444	2150
58.	*C.c.*	TLMF Lep 02710	PHLAC675-10	JF860237	-	Schmid J.	PCJS	24.V.2008	Switzerland	Riefawald/ Vals	46.6180	9.1940	1800

№	Species name[1]	Sample ID	Process ID	Genbank COI	Genbank H3	Collector	Collection[2]	Collection date	Country	Locality	Latitude	Longitude	Elevation (m)
59.	*C.c.*	CLV1739	GRSLO219-10	KM253778	-	Wieser C.	LMK	20.VI.2003	Italy	Sella di Grubia	46.3656	13.4164	1700
60.	*C.c.*	BC ZSM Lep 61455	FBLMZ604-12	KM253779	-	Lichtmannecker P.	ZSM	05.VI.2010	Germany	Linderhof, Saegertal oben	47.5691	10.9053	1110
61.	*C.c.*	DP09034	GRPAL024-10	KM253772	-	Larsen K.	PCJWdP	26.VI.1982	Norway	Kongsvold	62.2833	9.6167	1140
62.	*C.c.*	TLMF Lep 00939	PHLAB139-10	HQ968444	-	Huemer P.	TLMF	26.VI.1989	Austria	S-Schafgafall	47.0670	9.7670	2150
63.	*C.c.*	TLMF Lep 01932	PHLAB1132-10	HQ968344	KM253793	Huemer P.	TLMF	08.VII.2010	Austria	E Laguzalpe/ Marul	47.1933	9.9533	1680
64.	*C.c.*	TLMF Lep 01933	PHLAB1133-10	HQ968345	KM253792	Huemer P.	TLMF	08.VII.2010	Austria	E Laguzalpe/ Marul	47.1933	9.9533	1680
65.	*C.c.*	TLMF Lep 01934	PHLAB1134-10	HQ968346	KM253797	Huemer P.	TLMF	08.VII.2010	Austria	E Laguzalpe/ Marul	47.1933	9.9533	1680
66.	*C.c.*	TLMF Lep 01935	PHLAB1135-10	HQ968347	KM253802	Huemer P.	TLMF	08.VII.2010	Austria	E Laguzalpe/ Marul	47.1933	9.9533	1680
67.	*C.c.*	CLV5634	ISSIK149-14	KM253780	-	Huemer P.	TLMF	19.VII.1992	Italy	Alpi Orobie , Val d`Arera	45.9967	10.0017	2000
68.	*C.c.*	CLV5635	ISSIK150-14	KM253773	-	Huemer P.	TLMF	19.VII.1992	Italy	Alpi Orobie , Val d`Arera	45.9967	10.0017	2000
69.	*C.c.*	MM08522	LEFIE207-10	HQ570365	-	Mutanen T.	UO	29.VI.2003	Finland	Enontekioe	68.9970	20.7440	404
70.	*C.c.*	MM08523	LEFIE208-10	HQ570366	-	Mutanen T.	UO	29.VI.2003	Finland	Enontekioe	68.9970	20.7440	404
71.	*C.c.*	MM08573	LEFIE246-10	HM873980	-	Mutanen M.	UO	14.VI.2008	Finland	Rovaniemi	66.5050	25.6520	183
72.	*C.c.*	MM18091	LEFIK516-10	JN271968	-	Mutanen T.	UO	06.VII.2003	Finland	Enontekioe	69.0456	20.8554	460
73.	*C.c.*	NK315	ISSIK274	KM25378	KM253800	Massaro	MCSN	28.VII.2013	Italy	Colere (BG)	45.9850	10.3210	2000

№	Species name[1]	Sample ID	Process ID	Genbank COI	Genbank H3	Collector	Collec-tion[2]	Collection date	Country	Locality	Lati-tude	Longi-tude	Eleva-tion (m)
			-14	2		M.	B						
74.	*C.c.*	NK277	ISSIK141 -14	KM25377 1	KM253789	Rekelj J.	PCJR	24.VII.2009	Slovenia	Julijske Alpe, Kanin, Podi, Petra Skalarja	46.3491	13.4766	2250
75.	*C.c.*	-	-	-	-	Huemer P.	TLMF	08.VII.2010	Austria	E Laguzalpe/ Marul	47.1933	9.9533	1680
76.	*C.c.*	-	-	-	-	Huemer P.	TLMF	08.VII.2010	Austria	E Laguzalpe/ Marul	47.1933	9.9533	1680
77.	*C.c.*	-	-	-	-	Huemer P.	TLMF	08.VII.2010	Austria	E Laguzalpe/ Marul	47.1933	9.9533	1680
78.	*C.c.*	-	-	-	-	Huemer P.	TLMF	08.VII.2010	Austria	E Laguzalpe/ Marul	47.1933	9.9533	1680
79.	*C.c.*	-	-	-	-	Huemer P.	TLMF	26.VI.1989	Austria	S-Schafgafall	47.0667	9.76667	2150
80.	*C.c.*	-	-	-	-	Schmid J.	TLMF	24.V.2008	Switzerla nd	Riefawald/ Vals	46.6178	9.1935	1800
81.	*C.c.*	-	-	-	-	Zürnbauer F.	TLMF	26.IX.1967	Germany	Taubenberg	47.8167	11.7111	750
82.	*C.c.*	-	-	-	-	Mazzoleni F. & Massaro M.	MCSN B	28.VII.2013	Italy	Nido Aquila - rif. Albani	45.9661	10.0534	2000
83.	*C.c.*	-	-	-	-	Huemer P.	TLMF	01.VIII.2013	Italy	Sennes/ Seitenbachscharte (= Forcela de Riciogogn)	46.6725	12.0472	2300
84.	*C.c.*	-	-	-	-	Huemer P. & Tarmann G. M.	TLMF	19.VII.1992	Italy	Val d'Arera	45.9281	9.7925	2000

№	Species name[1]	Sample ID	Process ID	Genbank COI	Genbank H3	Collector	Collec-tion[2]	Collection date	Country	Locality	Lati-tude	Longi-tude	Eleva-tion (m)
85.	*C.c.*	-	-	-	-	Huemer P.	TLMF	01.VIII.2004	Austria	Arzler Scharte - Pfeishütte	47.3262	11.4212	1950
86.	*C.c.*	-	-	-	-	Erlebach S. & Huemer P.	TLMF	11.IX.1997	Austria	Äußere Ebenalm,Bretterbrugg	47.0331	12.7883	1640
87.	*C.c.*	-	-	-	-	Burmann K.	SMNK	10.VII.1941	Austria	Blockkogel	47.0863	10.8814	3097
88.	*C.c.*	-	-	-	-	Burmann K.	SMNK	12.VII.1941	Austria	Blockkogel	47.0863	10.8814	3097
89.	*C.c.*	-	-	-	-	Burmann K.	SMNK	11.VII.1941	Austria	Blockkogel	47.0863	10.8814	3097
90.	*C.c.*	-	-	-	-	Huemer P. & Schatz I. & Tarmann G.	TLMF	01.IX.1988	Austria	Daberklamm E/ Kals am Großglockner NW	47.0381	12.6247	1650
91.	*C.c.*	-	-	-	-	Tarmann G. M.	TLMF	29.VI.1988	Austria	Daberklamm N/ Kals am Großglockner NW	47.0325	12.6253	1600
92.	*C.c.*	-	-	-	-	Burmann K.	SMNK	10.VII.1941	Austria	Darmstädter Hütte	47.0532	10.2464	2384
93.	*C.c.*	-	-	-	-	Gradl F.	VND	10.VI.1934	Austria	Flexenpass	47.1500	10.1600	1700
94.	*C.c.*	-	-	-	-	Huemer P.	TLMF	25.VII.1999	Austria	Flexenpaß S	47.1500	10.1667	1750
95.	*C.c.*	-	-	-	-	Burmann K.	SMNK	data unknown	Austria	Franz Senn Hütte	47.0860	11.1698	2149
96.	*C.c.*	-	-	-	-	Huemer P.	TLMF	16.IX.1993	Austria	Gumpachkreuz/ Prägraten NW	47.0458	12.3342	1884
97.	*C.c.*	-	-	-	-	Gradl F.	VND	16.VI.1929	Austria	Hasenfluh W. Zürs	47.1600	10.1400	2500

№	Species name[1]	Sample ID	Process ID	Genbank COI	Genbank H3	Collector	Collec-tion[2]	Collection date	Country	Locality	Lati-tude	Longi-tude	Eleva-tion (m)
98.	*C.c.*	-	-	-	-	Burmann K.	SMNK	02.VII.1940	Austria	Höttinger Alm S - Innsbruck NW	47.2995	11.3677	1487
99.	*C.c.*	-	-	-	-	Süssner L.	TLMF	16.VII.1959	Austria	Kapall/ St.Anton am Arlberg NW	47.1495	10.2500	2333
100.	*C.c.*	-	-	-	-	Huemer P.	TLMF	23.VIII.1994	Austria	Moaralm/ Kals am Großglockner NW	47.03325	12.6296	1783
101.	*C.c.*	-	-	-	-	Burmann K.	SMNK	01.III.1970	Austria	Obergurgl	46.8711	11.0275	1927
102.	*C.c.*	-	-	-	-	Burmann K.	SMNK	01.IX.1969	Austria	Obergurgl	46.8711	11.0275	1927
103.	*C.c.*	-	-	-	-	Burmann K.	SMNK	01.VIII.1971	Austria	Obergurgl	46.8711	11.0275	1927
104.	*C.c.*	-	-	-	-	Burmann K.	SMNK	05.VII.1980	Austria	Obergurgl	46.8711	11.0275	1927
105.	*C.c.*	-	-	-	-	Wimmer J.	PCJW	03.VII.2001	Austria	Schaumbergalm, rückwärts	47.7797	14.4203	1050
106.	*C.c.*	-	-	-	-	Burmann K.	SMNK	05.VI.1941	Austria	Schlicker Alm/ Fulpmes W	47.1557	11.3035	1643
107.	*C.c.*	-	-	-	-	Wimmer J.	PCJW	05.VI.2003	Austria	Sender (Spering)	47.8017	14.2080	1482
108.	*C.c.*	-	-	-	-	Tarmann G. M.	TLMF	25.VI.1991	Austria	Teischnitztal/ Kals am Großglockner N	47.0352	12.6590	2000
109.	*C.c.*	-	-	-	-	Burmann K.	SMNK	26.VI.1955	Austria	Vennatal/ Brenner NE	47.0142	11.5342	1500
110.	*C.c.*	-	-	-	-	Burmann K.	SMNK	02.VII.1955	Austria	Vennatal/ Brenner NE	47.0142	11.5342	1500
111.	*C.c.*	-	-	-	-	Burmann K.	SMNK	03.VII.1955	Austria	Vennatal/ Brenner NE	47.0142	11.5342	1500
112.	*C.c.*	-	-	-	-	Burmann	SMNK	18.VI.1947	Austria	Vennatal/ Brenner	47.0142	11.5342	1500

№	Species name[1]	Sample ID	Process ID	Genbank COI	Genbank H3	Collector	Collec-tion[2]	Collection date	Country	Locality	Lati-tude	Longi-tude	Eleva-tion (m)
						K.				NE			
113.	*C.c.*	-	-	-	-	Burmann K.	SMNK	01.III.1956	Austria	Vent	46.8632	10.9176	1885
114.	*C.c.*	-	-	-	-	Gradl F.	VND	16.VI.1932	Austria	Zürs	47.1700	10.1600	1600
115.	*C.c.*	-	-	-	-	Gradl F.	VND	30.V.1934	Austria	Zürs	47.1700	10.1600	1600
116.	*C.c.*	-	-	-	-	Triberti P.	MCSN	20.III.1992	Italy	Colle Isarco Malga Zirago	46.9668	11.4960	1800
117.	*C.c.*	-	-	-	-	Triberti P.	MCSN	20.III.1992	Italy	Colle Isarco Malga Zirago	46.9668	11.4960	1800
118.	*C.c.*	-	-	-	-	Triberti P.	MCSN	20.III.1992	Italy	Colle Isarco Malga Zirago	46.9668	11.4960	1800
119.	*C.c.*	-	-	-	-	Triberti P.	MCSN	20.III.1992	Italy	Colle Isarco Malga Zirago	46.9668	11.4960	1800
120.	*C.c.*	-	-	-	-	Triberti P.	MCSN	20.III.1992	Italy	Colle Isarco Malga Zirago	46.9668	11.4960	1800
121.	*C.c.*	-	-	-	-	Triberti P.	MCSN	20.III.1992	Italy	Colle Isarco Malga Zirago	46.9668	11.4960	1800
122.	*C.c.*	-	-	-	-	Triberti P.	MCSN	20.III.1992	Italy	Colle Isarco Malga Zirago	46.9668	11.4960	1800
123.	*C.c.*	-	-	-	-	Klimesch J.	MCSN	26.III.1939	Austria	Warscheneck	47.6500	14.2300	420
124.	*C.c.*	-	-	-	-	Rocca L.	MCSN	04.VII.1933	Italy	A. Carniche, Sappada, Passo Siera	46.5433	12.6772	1600
125.	*C.c.*	-	-	-	-	Rocca L.	MCSN	02.VII.1933	Italy	A. Carniche, Sappada, L. d’Olbe	46.5929	12.6916	2000
126.	*C.c.*	-	-	-	-	Baldizzone G.	MCSN	27.VI.20VI	Italy	Valle d’Aosta Champorcher	45.6195	7.5631	2300

№	Species name[1]	Sample ID	Process ID	Genbank COI	Genbank H3	Collector	Collection[2]	Collection date	Country	Locality	Latitude	Longitude	Elevation (m)
127.	*C.c.*	-	-	-	-	Baldizzone G.	MCSN	27.VI.20VI	Italy	Valle d'Aosta Champorcher	45.6195	7.5631	2300
128.	*C.c.*	-	-	-	-	Karsholt O.	TLMF	21.VI.1981	Norway	Fokstua	62.1190	9.2840	950
129.	*C.c.*	-	-	-	-	Huemer P.	TLMF	04.VII.1983	Austria	Vorarlberg Brandner Tal Boser Tritt	47.0638	9.7515	1700
130.	*C.c.*	-	-	-	-	Huemer P.	TLMF	08.VII.1982	Austria	Vorarlberg Brandner Tal Boser Tritt	47.0638	9.7515	1700
131.	*C.c.*	-	-	-	-	Tarmann G.	TLMF	29.VI.1988	Austria	Glocknergruppe Daberklamm	47.0304	12.6395	1650
132.	*C.c.*	-	-	-	-	Tarmann G.	TLMF	29.VI.1988	Austria	Glocknergruppe Teischnitztal	47.0288	12.6293	1650
133.	*C.c.*	-	-	-	-	Burmann K.	TLMF	09.VII.1984	Austria	Bodenalpe	46.9500	10.2700	2000
134.	*C.c.*	-	-	-	-	Svensson I.	TLMF	13.VII.1975	Sweden	Bäno	66.6967	16.0872	700
135.	*C.c.*	-	-	-	-	Zürnbauer	TLMF	IX.1967	Germany	Taubenberg	47.8270	11.7315	750

*All listed samples were studied morphologically; the barcoded samples are supplied with Sample ID, Process ID, Genbank COI and Genbank H3 (if nuclear gene histone H3 was analyzed).

[1]**Species name:** *C.b.* – *Callisto basistrigella* Huemer, Deutsch & Triberti, sp. n., *C.c.* – *Callisto coffeella* (Zetterstedt 1839).

[2]**Collection:** LMK – Landesmuseum Kärnten; Klagenfurt, Austria; MCSN – Museo Civico di Storia Naturale, Verona, Italy; MCSNB –Museo Civico di Scienze Naturali "E. Caffi", Bergamo, Italy; SMNK – Staatliches Museum für Naturkunde, Karlsruhe, Germany; TLMF – Tiroler Landesmuseum Ferdinandeum, Innsbruck, Austria; UO – University of Oulu, Finland; VND – Inatura Erlebnis Naturschau Dornbirn, Austria; ZSM – Zoologische Staatssammlung, Munich, Germany. Private collections: PCHD – Helmut Deutsch, Bannberg, Assling, Tyrol, Austria; PCJC – Jurij Rekelj, Kranj, Slovenia; PCJS – Jürg Schmid, Illanz, Switzerland; PCJW – Josef Wimmer, Steyr, Austria; PCJWdP – Jurate and Willy de Prins, London, UK; PCSG – Stanislav Gomboc, Slovenia.
